# An equity analysis of health examination service utilization by women from underdeveloped areas in western China

**DOI:** 10.1371/journal.pone.0186837

**Published:** 2017-10-24

**Authors:** Yuyan Qian, Jianmin Gao, Zhongliang Zhou, Ju’e Yan, Yongjian Xu, Xiaowei Yang, Yanli Li

**Affiliations:** 1 School of Public Health, Health Science Center, Xi’an Jiaotong University, Xi’an, Shaanxi, People’s Republic of China; 2 School of Humanities and Social Sciences, Xi’an Jiaotong University, Xi’an, Shaanxi, People’s Republic of China; 3 School of Public Policy and Administration, Xi’an Jiaotong University, Xi’an, Shaanxi, People’s Republic of China; 4 School of Law, Linyi University, Linyi, Shandong, People’s Republic of China; James Cook Unviersity, AUSTRALIA

## Abstract

**Objective:**

This study sought to examine the sources of inequity in health examination service utilization by women from underdeveloped areas in western China.

**Methods:**

Based on data from the 5^th^ National Health Service Survey in Shaanxi province, women’s utilization of health examination services was examined according to gynecological, cervical smear, and breast examination rates. The equity of health examination service utilization by 15- to 64-year-old women and the factors contributing to inequity were determined using the health concentration index, decomposition of the concentration index, and the horizontal inequity index.

**Results:**

The examination rates for gynecological, cervical smear, and breast exams for 15- to 64-year-old women in Shaanxi province were 40.61%, 27.08%, and 24.59%, respectively. The horizontal inequity indices of gynecological, cervical smear, and breast examination rates were 0.0480, 0.0423, and 0.0764, respectively, and each examination rate was higher for wealthy individuals. The contribution rates of economic status to the inequalities in gynecological, cervical smear, and breast examination rates were 65.80%, 74.31%, and 56.49%, respectively. The contribution rates of educational status to the inequalities in gynecological, cervical smear, and breast examination rates were 21.01%, 14.83% and 30.00%, respectively. The contribution rates of age to the inequalities in gynecological, cervical smear, and breast examination rates were 25.77%, 26.55%, and 18.40%, respectively.

**Conclusions:**

Women’s health examination rates differed between populations with different socio-demographic characteristics. There is pro-wealth inequality in each examination rate. This study found that financial status, age, and education level were the main reasons for the unequal utilization of health examination services.

## Introduction

Health inequality refers to a special type of health disparity or a difference in important health determinants. In this difference, the vulnerable groups show a worse health status than the favorable group and therefore face greater health risks [1–4]. The Commission on Social Determinants of Health notes that within countries, dramatic differences in health are closely linked to the degrees of social determinants of health. These avoidable health inequalities arise because of the circumstances in which people grow up, live, work, and age as well as the systems put in place to deal with illness. In most countries, health and illness follow a social gradient at all levels of income: the lower the socioeconomic position, the worse an individual’s health tends to be. This unequal distribution of health-damaging experiences is usually combined with poor social policies and programs, unfair economic arrangements, and bad politics [5]. Therefore, measuring the equity of health, finding the source of inequity, and evaluating the impact of policy and action on health equity will contribute to reducing health inequity.

Previous studies have found that routine gynecological examination is an effective method for the early detection and prevention of gynecological diseases, which improves the quality of life for women [6]. Preventive examinations, which include the assessment of medical conditions and the diagnosis of disease symptoms in the preclinical phase, provide critical information for the prevention of disease and improve the effectiveness of drugs and rehabilitation in the event of an early diagnosis. In particular, preventive screening is one of the best ways to improve the negative epidemiological indicators of treatment outcomes for the two major life-threatening cancers in women: breast cancer and cervical cancer [7–10]. After examining the relationship between women’s benefits and disease burden, the most recent annual report from the American Cancer Society (ACS) indicated that the benefits of intervention outweigh the adverse effects; therefore, it is recommended that women receive regular breast and cervical cancer screenings [11]. A few recent studies have reported that economic status, educational status, geographical differences, ethnicity, age, attitude, and insurance participation are the main factors that influence women’s participation in health examinations [7, 12–19]. Furthermore, vulnerable groups still face unequal access to free screening programs provided by the government [20].

Several studies have reported inequity in the utilization of health examination services via regression analysis [21, 22], relative index [23], or slope index [24], but few studies have measured inequity via the concentration index and decomposition of the concentration index. Therefore, selected key indicators were used in this study to examine equity in health examination service utilization by women from underdeveloped areas in the western region of China. To the best of our knowledge, this study is the first to examine inequality in the utilization of health examination services using a large-scale representative female sample from Shaanxi province, which is located in a western economically underdeveloped area of China. Our findings will be of great importance in formulating and modifying public health policies, improving the inequality of health examination service utilization by women, and enhancing women’s health.

## Method

### Data source

The data in this study originated from the family health survey of the 5^th^ National Health Service Survey (henceforth NHSS) of Shaanxi province and it belongs to the Shaanxi Health and Family Planning Commission (SHFPC). Shaanxi province is located in the northwest region of China with a size of approximately 205,800 km^2^ and a resident population of 37.637 million, among which women account for 48.36% of the population [25]. As of 2013, the per capita GDP of Shaanxi province was 42,692 yuan, which makes it one of the more economically underdeveloped regions in China compared to the 62,405 yuan per capita GDP in the eastern regions of China [26]. A multistage, stratified, clustering, and randomized sampling method was used for the 5^th^ NHSS in Shaanxi province, in which 32 counties (districts) were randomly selected in the first stage, 160 towns within those counties or districts were selected in the second stage, 320 villages (communities) within those towns were selected in the third stage, and 20,700 families within those counties or districts were selected in the fourth stage [27, 28], for a final total of 57,529 individuals. Missing households that were not available after 3 contacts were replaced with alternative households based on a predefined protocol. A face-to-face household interview was conducted using a questionnaire developed by the Health Statistics and Information Center of the Ministry of Health of China. This questionnaire includes general information on the socioeconomic and demographic characteristics of the family, insurance, self-reported diseases and injuries as well as outpatient and inpatient healthcare utilization. For 15-64-year-old women, an additional survey on physical examinations, childbirth, and maternity care within the last year was also conducted. All data were collected from the face-to-face interviews, and the data on the three types of health service utilization were self-reported. The current study included 15- to 64-year-old women as the subjects of the study. After excluding households with invalid answers and/or with key variables missing, the final sample included 6,713 urban women and 12,121 rural women for a total of 18,834 women.

### Indicators for measuring health examination service utilization

Cervical cancer, breast cancer, and reproductive tract infections [19] are common diseases found in women. As the basic indicators of diagnosing these common diseases and available indicators in the NHSS, the cervical smear, breast exam, and gynecological exam were selected as indicators for assessing health examination service utilization by women in this study. The following definitions were applied:

Cervicalsmearrate=numberofwomenreceivingacervicalsmear/numberofwomensurveyed*100% (1)

Breastexaminationrate=numberofwomenreceivingabreastexamination/numberofwomensurveyed*100% (2)

Gynecologicalexaminationrate=numberofwomenreceivingagynecologicalexamination/numberofwomensurveyed*100% (3)

### Inequity analysis of health examination service utilization by women

#### Economic status

In previous studies, the per capita income or consumption expenditure was used as an indicator of economic status [29–31]. Although the annual household income and consumption expenditure are provided by the NHSS, it was suggested that the reporting bias of self-reported income was greater than the self-reported consumption expenditure, and the income of the surveyed individuals in developing countries may have been underestimated [3]. Therefore, in this study, the household consumption expenditure was used to measure economic status. In the NHSS, household consumption expenditure included 8 categories, specifically food, daily life, transportation and communication, housing, education, entertainment, health care, and other expenses. The results of this survey represented the total expenditure of family members on each category. Since household economies of scale are neglected when using the traditional method for calculating the per capita consumption expenditure (annual household consumption expenditure/number of household member), adjustments to the household economies of scale and age distribution were required during data analysis [32]. Given that children and adults have different needs, the household size should be adjusted to the number of adult equivalents [33], as shown by the following formula (4):

$$ΑΕ={\left(Α+\rho Κ\right)}^{\theta }$$ (4)

where *AE* is the adjusted number of household members, *A* is the number of individuals 15 years of age and older, *K* is the number of individuals 15 years of age and under, *ρ* is the minor coefficient (0.3 was used in this study for developing countries), and *θ* is the level of household economies of scale (generally 0.75–1, and in the present study, a value of 0.75 was used). After adjusting the number of household members per the above formula, the annual per capita consumption expenditure was calculated by the annual household consumption expenditure/ adjusted household size (i.e., the number of adult equivalents in each household).

#### Concentration index

A concentration index is the most scientific and effective method for measuring health equity [34]. In this study, we used the concentration index to analyze and compare the utilization of health examination services among women at different economic levels. The concentration index was calculated as follows:

C=2cov(x,h)∕μ (5)

where *x* is a ranking of economic level, *h* is a ranking of health examination service utilization, and μ is the average of health examination service utilization.

#### Decomposition of concentration index and horizontal inequity index

The concentration index of each factor was decomposed into the contribution of each factor to the unequal utilization of health examination service based on a probit regression model. Because the utilization of health examination services is a binary response, the probit regression model is used with the probability of gynecological examination, cervical smear examination, and breast examination as the dependent variables to indirectly standardize the health examination service utilization. The general functional form of such a nonlinear model can be written as follows:

$$y_{i}=a^{m}+\sum_{j}\beta_{j}^{m}x_{ji}+\sum_{k}\gamma_{k}^{m}z_{ki}+\mu_{i}$$ (6)

where y_*i*_ is the utilization of the health examination service, *x*_ji_ is the “need” variable, *z*_*ki*_ is the “control” variable, $\beta_{j}^{m}$ and $\gamma_{k}^{m}$ are the marginal effects of each variable evaluated at sample means, and *μ*_i_ is the error term.

The decomposition of the concentration index C could be specified as follows:

$$C=\sum_{j}(\beta_{j}^{m}{\bar{x}}_{j}∕\mu )C_{j}+\sum_{k}(\gamma_{k}^{m}{\bar{z}}_{k}∕\mu )C_{k}+GC_{\mu }∕\mu$$ (7)

where *C* is the non-standardized concentration index of *y*, *C*_*j*_ is the concentration index of *x*_*j*_, *C*_*k*_ is the concentration index of *γ*_*k*_, *GC*_*μ*_ is the concentration index of the residual term, and ${\bar{x}}_{j}$ and ${\bar{z}}_{k}$ are the average of *x*_*j*_ and *z*_*k*_, respectively. A positive contribution rate of a variable indicates that the variable increases the inequality of the results when it is the only impact factor, and vice versa. This method has been widely used to analyze the impact factors of health equity, and it can more accurately depict the distribution of inequality in health examination service utilization under different socioeconomic conditions [33, 35, 36].

As shown in the formula, the concentration index *C* of health examination service utilization is equal to the weighted sum of the concentration index of “need” variables and “control” variables (the residual term is not considered). The NHSS is a census of the general population according to the literature [33,37–39], and based on the availability of the data, we selected age, illness in the last two weeks, chronic disease, and self-rated health as the need variables. The “control” variables included education status, marital status, employment status, economic status, urban, rural, and region (see Table 1). The product of each "need" variable and "control" variable concentration index and its weighting indicate the contributions of these variables to the inequality of health examination service utilization. Subtraction of the “need” variable contribution from the concentration index of health examination service utilization produces the horizontal inequity index [4, 40].

**Table 1:** Description of independent variables.

		N	Percent (%))
Age (years)	15–24*	2,222	11.80
	25–34	2,954	15.68
	35–44	4,576	24.30
	45–54	5,167	27.43
	55–64	3,915	20.79
Education status	Illiterate*	2,875	15.26
	Primary school	4,526	24.03
	Junior high school	7,388	39.23
	Senior high school	2,708	14.38
	Junior college and above	1,337	7.10
Marital status	Unmarried*	1,953	10.37
	married	16,128	85.63
	Widowed and divorced	753	4.00
Employment status	Employed*	14,503	77.00
	retirement	658	3.49
	student	906	4.81
	unemployed	2,767	14.69
Economic status	Lowest group*	3,885	20.63
	Lower group	3,650	19.38
	Medium group	3,769	20.01
	Higher group	3,764	19.99
	Highest group	3,766	20.00
Urban and rural	Urban*	6,713	35.64
	rural	12,121	64.36
Region	Shannan*	6,042	32.08
	Guanzhong	9,646	51.22
	Shanbei	3,146	16.70
Illness in last two weeks	Not ill in last two weeks*	15,463	82.10
	Ill in last two weeks	3,371	17.90
Chronic disease	No chronic disease*	15,209	80.75
	Has chronic disease	3,625	19.25
Self-rated health	Good*	14,492	76.95
	Poor	4,342	23.05

* Reference levels in the probit regression

### Statistical analysis

A Chi-square test was used to analyze the differences in the women's health examination service utilization for different social-demographic characteristics. The significance level was 0.05. All statistical analyses were performed using Stata13.0 software, and all tables and figures were made using Excel 2013.

### Ethical review

All investigators in this study obtained verbal informed consent from the participants. Before the survey was conducted, the Shaanxi Health and Family Planning Commission issued a governmental document asking for cooperation from the sample counties. Officials from the sample counties would contact each participant to obtain his or her verbal informed consent before administering the survey. If an individual agreed to participate in the interview, the official would make an appointment with that individual. Then, the investigators would go to the participant’s house to administer the survey.

This study was approved by the Ethics Committee of the Health Science Center of Xi’an Jiaotong University and complied with the ethical principles in the Declaration of Helsinki (Approval number: 2015–644).

## Results

### Health examination service utilization by women

The gynecological, cervical smear, and breast examination rates of 15- to 64-year-old women in Shaanxi province were 40.61%, 27.08%, and 24.59%, respectively. Among women of different ages, the 35- to 44-year-old group showed the highest rate of health examination service utilization. The group with primary school education showed the highest gynecological and cervical smear examination rates, while the group educated at junior college and above showed the highest breast examination rate. The examination rate in the married group was higher compared to the other groups. The gynecological and cervical smear examination rates in the employed group and the breast examination rate in the retired group were higher compared to those in groups with other employment statuses. The gynecological examination rate was higher in groups with a higher economic status. The cervical smear rate was lower, while the breast examination rate was higher, in urban women compared to rural women. The results showed no significant difference in gynecological examination rates between urban and rural women. Women in the Shannan region showed the highest gynecological and cervical smear examination rates, whereas women in the Guanzhong region showed the highest breast examination rate. Women who were ill in last two weeks or had chronic diseases showed higher rates of cervical smear examinations than women without illness, although no significant differences were observed for the gynecological and breast examination rates between women who were ill in the last two weeks or who had chronic disease and those who were not ill in the last two weeks or who had no chronic disease. The women who had self-rated poor health showed higher rates of gynecological and breast examinations than women with self-rated good health, although the cervical smear examination rate did not differ between women with self-rated good health and those with self-rated poor health (see Table 2).

**Table 2:** Utilization of health examination services.

	Gynecological examination rate (%)	χ^2^	P	Cervical smear examination rate (%)	χ^2^	P	Breast examination rate (%)	χ^2^	P
Age group		1,200	0.000		758.317	0.000		676.317	0.000
15–24	16.65			7.56			8.69		
25–34	49.02			26.81			28.44		
35–44	52.82			36.10			33.19		
45–54	44.46			32.20			27.75		
55–64	28.53			21.05			16.48		
Educational status		72.517	0.000		77.850	0.000		140.468	0.000
Illiterate	35.86			28.45			18.78		
Primary school	42.89			31.37			23.24		
Junior high school	42.35			25.41			25.61		
Senior high school	36.00			23.04			25.04		
Junior college and above	42.86			27.00			35.08		
Marital status		961.062	0.000		549.686	0.000		464.361	0.000
Unmarried	8.70			5.12			5.53		
Married	44.83			29.93			27.23		
Widowed and divorced	33.07			22.97			17.40		
Employment status		636.757	0.000		391.098	0.000		321.858	0.000
Employed	44.31			29.86			26.69		
Retirement	34.65			21.58			28.72		
Student	3.97			1.99			1.99		
Unemployed	34.66			22.05			19.99		
Economic status		148.616	0.000		76.193	0.000		168.117	0.000
Lowest group	34.59			23.42			19.79		
Lower group	39.64			27.10			23.32		
Medium group	39.53			26.00			23.24		
Higher group	41.42			26.91			24.65		
Highest group	48.04			32.08			32.05		
Urban and rural		0.522	0.470		8.628	0.003		54.200	0.000
Urban	40.27			25.80			27.69		
Rural	40.81			27.79			22.87		
Region		226.382	0.000		354.191	0.000		147.457	0.000
Shannan	48.30			35.83			25.47		
Guanzhong	37.72			23.62			26.78		
Shanbei	34.74			20.88			16.18		
Illness in last two weeks		0.055	0.815		4.247	0.039		1.980	0.159
Not ill in last two weeks	40.65			26.77			24.79		
Ill in last two weeks	40.43			28.51			23.64		
Chronic disease		0.269	0.604		29.869	0.000		0.005	0.943
No chronic disease	40.52			26.21			24.58		
Has chronic disease	40.99			30.70			24.63		
Self-rated health		14.050	0.000		0.011	0.915		14.152	0.000
Good	41.35			27.10			25.23		
Poor	38.16			27.02			22.43		

### Inequity in women’s utilization of health examination services

Table 3 shows the concentration indices of all indicators of women’s health examination service utilization. The concentration indices of gynecological, cervical smear, and breast examination rates were 0.0652, 0.0580, and 0.0940, respectively. All values were positive, which indicated a pro-wealth inequality in health examination service utilization by women.

**Table 3:** Concentration index (CI) of utilization of health examination services.

	CI	Std.	95% Confidence interval	
Gynecological examination rate	0.0652	0.0051	0.0552	0.0752
Cervical smear examination rate	0.0580	0.0069	0.0444	0.0716
Breast examination rate	0.0940	0.0074	0.0794	0.1085

The decomposition of the concentration index of each health examination service utilization indicator is shown in Table 4. The contribution rate is the percentage of each variable’s contribution to the inequality of gynecological, cervical smear, and breast examination rates. Economic status was found to have the highest contribution to the inequality of the three examinations, specifically 65.80%, 74.31%, and 56.49%, respectively, and the contribution rate increased with the economic level. Age and education level were found to be the second largest contributions to inequality. The contribution rate of age to the inequalities in gynecological, cervical smear, and breast examination rates were 25.77%, 26.55%, and 18.40%, respectively. The 35- to 44-year-old group showed the greatest contribution to these inequalities. The contribution rates of educational status to the inequalities in gynecological, cervical smear, and breast examination rates were 21.01%, 14.83%, and 30.00%, respectively. The contribution rates increased as educational status increased, with the junior college and above group showing the greatest contribution rates.

**Table 4:** Concentration index decomposition of utilization of health examination services.

	Gynecological examination rate				Cervical smear examination rate				Breast examination rate			
	dF/dx	Elasticity	Contribution	%	dF/dx	Elasticity	Contribution	%	dF/dx	Elasticity	Contribution	%
Age (years) (Reference level = 15–24)				25.77				26.55				18.40
25–34	0.053***	0.0409	0.0027	4.14	0.055***	0.0634	0.0042	7.24	0.039***	0.0496	0.0033	3.51
35–44	0.041***	0.0736	0.0071	10.89	0.059***	0.1592	0.0153	26.38	0.040***	0.1188	0.0114	12.13
45–54	0.012*	0.0332	0.0000	0.00	0.037***	0.1491	0.0002	0.34	0.019***	0.0860	0.0001	0.11
55–64	-0.019***	-0.0489	0.0070	10.74	0.008*	0.0297	-0.0043	-7.41	-0.004	-0.0176	0.0025	2.66
Education status (Reference level = Illiterate)				21.01				14.83				30.00
Primary school	0.013	0.0078	-0.0008	-1.23	-0.009	-0.0080	0.0008	1.38	0.000	-0.0004	0.0000	0.00
Junior high school	0.013	0.0122	0.0000	0.00	-0.043***	-0.0627	-0.0002	-0.34	0.011	0.0174	0.0001	0.11
Senior high school	0.048**	0.0170	0.0030	4.60	-0.001	-0.0006	-0.0001	-0.17	0.051***	0.0296	0.0051	5.43
Junior college and above and above	0.158***	0.0277	0.0115	17.64	0.075***	0.0196	0.0081	13.97	0.191***	0.0552	0.0230	24.47
Marital status (Reference level = Unmarried)				1.23				0.69				0.64
Married	0.331***	0.6974	0.0040	6.13	0.188***	0.5959	0.0034	5.86	0.182***	0.6325	0.0036	3.83
Widowed and divorced	0.352***	0.0346	-0.0032	-4.91	0.216***	0.0319	-0.0030	-5.17	0.202***	0.0328	-0.0030	-3.19
Employment status (Reference level = Employed)				-4.29				-6.90				-1.60
Retirement	-0.033	-0.0028	-0.0012	-1.84	-0.044*	-0.0056	-0.0024	-4.14	0.006	0.0009	0.0004	0.43
Student	-0.231***	-0.0274	-0.0016	-2.45	-0.154***	-0.0273	-0.0016	-2.76	-0.164***	-0.0321	-0.0019	-2.02
Unemployed	-0.068***	-0.0246	0.0009	1.38	-0.057***	-0.0310	0.0011	1.90	-0.039***	-0.0235	0.0008	0.85
Economic status (Reference level = Lowest group)				65.80				74.31				56.49
Lower group	0.042***	0.0203	-0.0080	-12.27	0.036***	0.0257	-0.0101	-17.41	0.025*	0.0201	-0.0080	-8.51
Medium group	0.030*	0.0146	0.0000	0.00	0.017	0.0126	0.0000	0.00	0.018	0.0145	0.0000	0.00
Higher group	0.046***	0.0227	0.0091	13.96	0.029**	0.0214	0.0085	14.66	0.024*	0.0193	0.0077	8.19
Highest group	0.106***	0.0522	0.0418	64.11	0.076***	0.0559	0.0447	77.07	0.082***	0.0668	0.0534	56.81
Urban and rural (Reference level = Rural)				-7.82				-7.41				3.62
Urban	-0.029***	-0.0253	-0.0051	-7.82	-0.016*	-0.0215	-0.0043	-7.41	0.012	0.0170	0.0034	3.62
Region (Reference level = Shannan)				-6.60				-7.93				-13.30
Guanzhong	-0.120***	-0.1517	0.0071	10.89	-0.119***	-0.2246	0.0104	17.93	-0.006	-0.0117	0.0005	0.53
Shanbei	-0.160***	-0.0658	-0.0114	-17.48	-0.140***	-0.0865	-0.0150	-25.86	-0.111***	-0.0751	-0.0130	-13.83
Illness in last two weeks (Reference level = Not sick)				-0.15				-0.69				-0.43
Ill in last two weeks	-0.005	-0.0022	-0.0001	-0.15	-0.017	-0.0113	-0.0004	-0.69	-0.017	-0.0123	-0.0004	-0.43
Chronic disease (Reference level = Not sick)				-0.15				-0.17				-0.11
Has chronic disease	0.036**	0.0170	-0.0001	-0.15	0.050***	0.0352	-0.0001	-0.17	0.034***	0.0265	-0.0001	-0.11
Self-rated health (Reference level = Good)				0.92				1.38				0.85
Poor	-0.023*	-0.0131	0.0006	0.92	-0.019*	-0.0165	0.0008	1.38	-0.017*	-0.0160	0.0008	0.85

* P<0.05

** P<0.01

*** P<0.001

The horizontal inequity indices of the gynecological, cervical smear, and breast examination rates after controlling for the effect of “need” variables on health examination service utilization were 0.0480, 0.0423, and 0.0764, respectively (Table 5). This finding reveals a pro-wealth inequity in health examination service utilization, and the horizontal inequity of the breast examination rate was higher than that of the gynecological and cervical smear examination rates.

**Table 5:** Horizontal inequity of utilization of health examination services.

	Gynecological examination rate	Cervical smear examination rate	Breast examination rate
Need variables	0.0172	0.0157	0.0176
Control variables	0.0461	0.0403	0.0721
Residual	0.0019	0.0020	0.0043
Total	0.0652	0.0580	0.0940
Horizontal inequity	0.0480	0.0423	0.0764

## Discussion

Our findings showed that 30- to 44-year-old women had the highest health examination service utilization rates, which may be due to the high incidence of common gynecological diseases [41] and the concentration of current cancer screenings within this age interval [13]. In addition, most women in this age group were married and employed and therefore had a higher need and better financial capability for health examination service. The trends in health examination service utilization among women of different educational statuses and geographic areas were consistent with the incidence of common gynecological diseases. Reproductive system diseases and cervical cancers are often found in rural populations and women with low education rates [42], although the incidence of breast cancer in cities is twice the rate in rural areas [43]. In addition, the coverage of cervical cancer screening in rural areas is greater than the coverage of breast cancer screening in the National Cancer Screening Program. As a result, the utilization of gynecological examinations and the cervical smear test were higher in rural women with less education, whereas the utilization of breast examination was higher in urban women with more education. The general trend of health examination service utilization was that the utilization increased as the economic level increased.

The concentration indices of gynecological, cervical smear, and breast examination rates were all positive, which indicated that the utilization of these tests by 15- to 64-year-old women was concentrated among the wealthiest women. Additionally, the breast examination rate showed the highest concentration index and greatest inequality among the three examinations.

Economic status showed the greatest contribution to inequality in gynecological, cervical smear, and breast examination rates. The elevation in economic status increased the pro-rich inequality in health examination service utilization and was consistent with the findings of other recent international studies [20, 22, 23]. Enhanced economic status increases health needs and health examination service utilization, and women with higher economic levels are more likely to take the initiative to access women's health examination services [40]. Additionally, women with higher economic status possess more financial capability in utilizing health examination services compared to women with a lower economic status. Together, these findings suggest that reducing the rich-poor gap is an essential solution to improving unequal health examination service utilization.

Education level increases pro-rich inequality in health examination service utilization. For instance, the education level is positively correlated with the overall health status in that higher levels of education increase the efficiency of producing healthy human capital and reduce the shadow price of health, which results in increased health flow and stock [44]. Therefore, women with higher education levels are not only more likely to have the economic conditions for accessing health examination services but also have a higher awareness of health and are therefore more proactive about acquiring health examination services.

Age is also a major factor that increases pro-wealth inequality in the utilization of health examination services, with the 30- to 44-year-old age group showing the greatest contribution rate. Overall, 30- to 44-year-old women of childbearing age have higher demands for health examination services. Most of these individuals are employed, and individuals with more employment benefits are enrolled in group physical examinations organized by their department every 1–2 years [11]. In fact, women who participated in group physical examinations accounted for more than 95% of women in Shaanxi province, which implied that women with greater utilization of health examination services in this age group had better incomes. As a result, this effect further increased the pro-wealth inequality in the utilization of health examination services.

Even though differences in socioeconomic conditions can lead to differences in the utilization of health examination services, they do not necessarily and adequately reflect the inequalities of accessing health services. To measure socioeconomic status-related inequality in health examination service utilization, health examination services must be standardized among the population. Measuring the inequalities in the utilization of health examination services among individuals with the same health examination service needs and different socioeconomic status is known as horizontal inequity. After removing the effect of the “need” variable, the horizontal inequity index showed that the pro-wealth inequality in utilization of health examination services was still present, and the horizontal inequity of the breast examination rate was higher compared to the gynecological and cervical smear examination rates.

It is important to note that although the difference between urban and rural areas was an important factor contributing to health examination service utilization inequality in past studies [19], its contribution was very small in the current study. This finding was mainly due to the benefits of the “Mother Health Project” developed in Shaanxi province [45], as well as the public health projects with free cancer screenings for rural women implemented by the government [46]. The “Mother Health Project” was implemented in Shaanxi province in 2008, and every two years per cycle, this program provides free examinations to 20- to 49-year-old married, childbearing women in rural areas. The program also provides active treatments to individuals with diseases based on the regulations for government subsidies. Additionally, the Chinese government implemented a nationwide “two-cancer” screening program for rural women in 2009 along with annual expansion of screening coverage. These free examination programs have greatly increased the health examination rates of rural women, which indicates that favorable health public policies can effectively improve inequalities in the utilization of health examination services.

This study has several limitations. First, since the data were drawn from Shaanxi province, the conclusion of this study may not be generalizable to China as a whole. Second, owing to data availability, not all factors that may impact the utilization of health examination services were considered in this study, such as women's attitudes towards health examinations, knowledge about health examinations and experience with health examinations. Furthermore, patients with breast cancer, cervical cancer and other gynecological diseases tend to have more health examinations, but this study did not take into account the high-risk factors for these patients; thus, the equity in this special population's health examination utilization may be different from the results of this study. Third, the impact of inequity on health examination service utilization is a long-term process. Thus, comparisons over time should be conducted for a more complete analysis of health examination service utilization.

## Conclusion

Similar to inequalities in other developing countries, inequalities in the utilization of health examination services are still common among women in Shaanxi province, and economic status is the major cause of these inequalities. However, in contrast to previous findings, the contributions of urban versus rural factors were insignificant in the current study mainly because of the benefits of the nation’s public health policies. Our results demonstrate that in addition to enhancing women’s economic and educational status and increasing women’s access to social resources, developing targeted national health policies and increasing access to free health examination service programs for low-income and elderly populations will help reduce inequalities in the utilization of health examination services.

## Supporting information

S1 File Family Health Questionnaire (Chinese version). Family health questionnaire developed by the Health Statistics and Information Center of the Ministry of Health of China. This questionnaire includes general information on the socioeconomic and demographic characteristics of the family, insurance, self-reported diseases and injuries as well as outpatient and inpatient healthcare utilization.
(DOCX)

S2 File Family Health Questionnaire (English version). This is the English version of Family Health Questionnaire. The content of this version is consistent with the Chinese version.
(DOCX)

## References

[1] Braveman P. Health disparities and health equity: concepts and measurement. Annual of Review Public Health. 2006; 27: 167–194. 10.1146/annurev.publhealth.27.021405.102103 16533114

[2] Wagstaff A. The Economic Consequences of Health Shocks: Evidence from Vietnam. Health Economics. 2007; 26(1):82–100. 10.1016/j.jhealeco.2006.07.001 16905205

[3] Zhou ZZ, Gao JM, Fox A, Rao KQ, Xu K, Xu L, et al Measuring the equity of inpatient utilization in Chinese rural areas. BMC Health Services Research. 2011; 11:201 doi: 10.1186/1472-6963-11-201 21854641 10.1186/1472-6963-11-201 PMC3271237

[4] Zhou ZZ, Gao JM, Xue QX, Yang XW, Yan JE. Effects of rural mutual health care on outpatient service utilization in Chinese village medical institutions: evidence from panel data. Health Econ. 2009; 18: S129–S136. doi: 10.1002/hec.1519 19548324 10.1002/hec.1519

[5] World health organization. Closing the gap in a generation: health equity through action on the social determinants of health. Available: http://apps.who.int/iris/bitstream/10665/43943/1/9789241563703_eng.pdf. 10.1016/S0140-6736(08)61690-6 18994664

[6] Wei WX, Wang Y, Li LY. Analysis of Gynecological Diseases among 10679 Women Participating in Physical Examination. Journal of Clinical Psychosomatic Diseases. 2016; 22(s2):64–65.

[7] Pilewkakozak AB, Klusek CŁ, Stadnicka G, Syty K, Kozak Ł, Jakiel G. Conditions of attending prophylactic gynecological examinations by women. Polish Journal of Public Health. 2014; 124(3):115–119.

[8] Mauad EC, Nicolau SM, Moreira LF, Haikel RL Jr, Longatto-Filho A, Baracat EC. Adherence to cervical and breast cancer programs is crucial to improving screening performance. Rural Remote Health. 2009; 9(3):1241 19778158

[9] Mierzwa T, Leźnicka M, Grodzki L, Kowalski W. Evaluation of the actions taken in order to increase the number of women undergoing preventive mammography and Pap smear in Kujawsko-Pomorskie province. Onkologia I Radioterapia. 2011; 17(3):47–60.

[10] Zhou KY, Xiang Y, An GM, Hu YH, Zhang JQ, Lu Z. Women’s health physical examination in Shaanxi Province. Chin Prev Med. 2016; 4:257–259.

[11] Smith RA, Andrews K, Brooks D, DeSantis CE, Fedewa SA, Tieulent JL, et al Cancer Screening in the United States, 2016: A Review of Current American Cancer Society Guidelines and Current Issues in Cancer Screening. CA Cancer J Clin. 2016; 66:95–114. 10.3322/caac.21336 26797525

[12] Carney PA, Goodrich ME, Mackenzie T, Weiss JE, Poplack SP, Wells WS, et al Utilization of screening mammography in New Hampshire. Cancer. 2005; 104(8):1726–1732. doi: 10.1002/cncr.21365 16158386 10.1002/cncr.21365

[13] Rada C, Prejbeanu I, Manolescu S. Attitudes to and practice of breast and cervical cancer screening in Romania. International Journal of Pharmacy Teaching & Practices. 2011; 2(1):49–56.

[14] Meng RL, Ma WJ, Xu YJ, Song XL, Nie SP, Xu HF, et al Screening rates of cervical cancer, breast cancer and the influential factors in Guangdong Province. South China Journal of Preventive Medicine. 2010; 2:8–10.

[15] Mu HJ, Yu LY, Li YX, Liu L, Zhan XM, Meng FF, et al Screening rates of cervical cancer, breast cancer and the influential factors among urban and rural residents in Liaoning Province. Chinese Journal of Public Health Management. 2015; 2:197–198.

[16] Gong PX. Screening rates of women’s cervical and breast cancer and their influential factors in Wenling. Chinese Journal of Public Health Management. 2015; 2:264–265.

[17] Yue DH, Hou ZY, Zhu YH, Fang H. Utilization and equity of preventive care in China from 1991 to 2011. Chinese Journal of Health Policy. 2015; 8(3):56–59.

[18] Coughlin SS, Thompson TD, Hall HI, Logan P, Uhler RJ. Breast and cervical carcinoma screening practices among women in rural and non-rural areas of the United States, 1998–1999. Cancer. 2002; 94(11):2801–12. doi: 10.1002/cncr.10577 12115366 10.1002/cncr.10577

[19] Zheng RM, Li X, Wang LH, Li LJ, Di JL, Fang LW. An Analysis of the Utilization of Census Service for Common Diseases among Chinese Women. Chinese Journal of Woman and Child Health Research. 2011; 22(6):798–801.

[20] Deandrea S, Molina-Barceló A, Uluturk A, Moreno J, Neamtiu L, Peiró-Pérez R, et al Presence, characteristics and equity of access to breast cancer screening programmes in 27 European countries in 2010 and 2014. Results from an international survey. Preventive Medicine. 2016; 91:250–263. doi: 10.1016/j.ypmed.2016.08.021 27527575 10.1016/j.ypmed.2016.08.021

[21] Couture MC, Nguyen CT, Alvarado BE, Velasquez LD, Zunzunegui MV. Inequalities in breast and cervical cancer screening among urban Mexican women. Preventive Medicine. 2008; 47(5):471–476. doi: 10.1016/j.ypmed.2008.07.005 18675296 10.1016/j.ypmed.2008.07.005

[22] Latasa P, Gandarillas AM, Ordobás M. Trends and social inequalities in cervical cancer and breast cancer screening in Madrid: Non-Commumicable Disease Risk Factor Surveillance System (SIVFRENT-A) from 1995 to 2010. Anales Del Sistema Sanitario De Navarra. 2015; 38(1):21–31. 25963455 10.23938/ASSN.0050

[23] Espinas JA, Aliste L, Fernandez E, Argimon JM, Tresserras R, Borras JM. Narrowing the equity gap: the impact of organized versus opportunistic cancer screening in Catalonia (Spain). J Med Screen. 2011; 18:87–90. doi: 10.1258/jms.2011.010086 21852701 10.1258/jms.2011.010086

[24] Chiou S, Wu C, Hurng B, Lu T. Changes in the magnitude of social inequality in the uptake of cervical cancer screening in Taiwan, a country implementing a population-based organized screening program. International Journal for Equity in Health. 2014; 13(1):1–8. 24405587 10.1186/1475-9276-13-4 PMC3896803

[25] Statistical Communique of Shaanxi Province on National Economic and Social Development. Available: http://www.shaanxi.gov.cn/0/1/65/365/369/169890.htm

[26] 2013 China Statistical Yearbook. Available: http://www.stats.gov.cn/tjsj/ndsj/2014/indexch.htm.

[27] Xu YJ, Gao JM, Zhou ZZ, Xue QX, Yang JJ, Luo H, et al Measurement and explanation of socioeconomic inequality in catastrophic health care expenditure: evidence from the rural areas of Shaanxi Province. BMC Health Services Research. 2016; 15:256. 10.1186/s12913-015-0892-2 PMC4490607 26138738

[28] Zhou ZZ, Gao JM. Study of catastrophic health expenditure in China’s basic health insurance. Health MED. 2011; 5:1498–507.

[29] Hu L. A demonstration study on the unfair health condition with their income in China. Health Economics Research. 2005; 12(04):13–16.

[30] Humphriesa K.H., Doorslaer EV. Income-related health inequality in Canada. Social Science & Medicine. 2000; 50:663–671. 10658847 10.1016/s0277-9536(99)00319-6

[31] Barraza-Lloréns M, Panopoulou G, Díaz BY. Income-related inequalities and inequities in health and health care utilization in Mexico, 2000–2006. Revista Panamericana De Salud Pública. 2013; 33(2):122–130. 23525342 10.1590/s1020-49892013000200007

[32] Chen L, Wu Y, Coyte PC. Income-related children’s health inequality and health achievement in China. International Journal for Equity in Health. 2014; 13(1):102. 25359568 10.1186/s12939-014-0102-6 PMC4229621

[33] Zhou ZZ, Su YF, Gao JM, Campbell B, Zhu ZW, Xu L, et al Assessing equity of healthcare utilization in rural China: results from nationally representative surveys from 1993 to 2008. International Journal for Equity in Health. 2013; 12: 34 doi: 10.1186/1475-9276-12-34 23688260 10.1186/1475-9276-12-34 PMC3673871

[34] Doorslear VE, Wagstaff A, Paci P. On the measurement of inequity in health. Soc Sci Med. 1991; 33(5): 545 1962226 10.1016/0277-9536(91)90212-u

[35] Asada Y. Assessment of the health of Americans: the average health-related quality of life and its inequality across individuals and groups. Population Health Metrics. 2005; 3(1): 7. 16014174 10.1186/1478-7954-3-7 PMC1192818

[36] Zhou ZZ, Zhu L, Zhou ZY, Li ZY, Gao JM, Chen G. The effects of China’s urban basic medical insurance schemes on the equity of health service utilization: evidence from Shaanxi province. International Journal for Equity in Health. 2014; 13:23 doi: 10.1186/1475-9276-13-23 24606592 10.1186/1475-9276-13-23 PMC4016277

[37] O’Donnell O, Doorslaer EV, Wagstaff A, Lindelow M. Analyzing Health Equity Using Household Survey Data. The World Bank Washington, D.C. 2008; 177–185.

[38] Wagstaff A, Doorslaer EV. Measuring and Testing for Inequity in the Delivery of Health Care. The Journal of Human Resources. 2000;35(4): 716–733.

[39] Remenschneider AK, Amico LD’, Gray ST, Holbrook EH, Gliklich RE, Metson R. The EQ-5D: A New Tool for Studying Clinical Outcomes in Chronic Rhinosinusitis. Laryngoscope. 2015;125(1):7–15. doi: 10.1002/lary.24715 24729050 10.1002/lary.24715

[40] Zhou ZZ, Fang Y, Zhou ZY, Li D, Wang D, Li YL, et al Assessing income-related health inequality and horizon inequity in China. Social Indicators Research. 2016; DOI: doi: 10.1007/s11205-015-1221-1

[41] Gao KY, Du ZQ, Xie ZH, Qiu ZQ, Yang LC. Analysis of 59178 Cases of Married Women’s Health Examination. China Foreign Medical Treatment. 2009; 36:138–140.

[42] Li TF, Chen RG. Overview of clinical epidemiology of cervical cancer. Practical Journal of Clinical Medicine. 2005; 2(2):19–22.

[43] Fan L, Strasser-Weippl K, Li JJ, St Louis J, Finkelstein DM, Yu KD, et al Breast cancer in China. The Lancet Oncology. 2014; 15(7):e279–89. doi: 10.1016/S1470-2045(13)70567-9 24872111 10.1016/S1470-2045(13)70567-9

[44] Mao Y, Feng GF. A Study on the Effect of Education on Health and Its Transmission Mechanism. Population & Economics. 2011; 3(186):87–93.

[45] Health and Family Planning Commission of Shaanxi. Investigation and research of "eugenics promotion project" and "mother health project" by Shaanxi province family planning guidance institute. Available: http://www.sxwjw.gov.cn/newstyle/pub_newsshow.asp?id=1052961&chid=100240.

[46] The document of management plan of cervical cancer and breast cancer screening project among rural women. Available: http://www.nhfpc.gov.cn/zwgkzt/wsbysj/200906/41534.shtml.

