# Supplementary material for: An equity analysis of health examination service utilization by women from underdeveloped areas in western China
Source: PLoS One. 2017 Oct 24;12(10):e0186837. doi: 10.1371/journal.pone.0186837 (PMC5655443; doi:10.1371/journal.pone.0186837)
Supplement: S2 File — This is the English version of Family Health Questionnaire. The content of this version is consistent with the Chinese version. (DOCX) [file pone.0186837.s002.docx]

**Appendix**

**Number: National Health Service Survey 1
Establishment of organs: National Health and Family Planning Commission
Approved Agency: National Bureau of Statistics
Approval Number: National Control [2013] No. 65
Expiration: December, 2013**

**Family Health Questionnaire**

**Address：__________County（City/District）_________Town（Street）_________Village（Residents’ Committee） ___________________________（Detailed Address）**

**Householder：_____________ Phone：**

**County（City/District）Administrative division code□□□□□□ Town（Street）code□□□ Village（Residents’ Committee）code□□□**

**Household code□□**

**Start time: :**  **am/pm / /2013**

**End time: :**  **am/pm / /2013 Investigator（Signature）：**

**Verification Date： / /2013** **Survey instructor（Signature）：**

**Guidance**

Dear Residents:
Hello! We are the investigator of the fifth National Health Service Survey. The survey was organized by the National Health Administration, and the contents of the survey were approved by the National Bureau of Statistics. The main purpose of the National Health Service Survey is to understand the health status of residents and the utilization of medical and health services, and to provide information for the state to formulate health policies and improve the health of the residents. All the contents of the survey are for statistical analysis only. We will keep the information of you and your family in accordance with the relevant provisions of the Statistical Law of the People's Republic of China. I hope you can answer the following questions honestly, thank you for your support and cooperation!

"Any information obtained in the survey that can identify or infer the identity of a single survey object shall not be provided or disclosed by any entity or individual, and it also shall not be used for purposes other than statistics.

Statistical Law of the People's Republic of China (Chapter III, Article 25)

List of questionnaires

Table 1. Household general situation questionnaire
Table 2. Family members' personal situation questionnaire
Table 3. Survey of injuries and illness in the last two weeks
Table 4. Survey of hospitalizations in the last one year
Table 5. Questionnaire for Children under 5 years of age
Table 6. Questionnaire for women aged 15-64 years

**Table 1. Household general situation questionnaire**

This table is answered by the person most familiar with the family situation in the surveyed household

| SN | Questions and options | Answer |
| --- | --- | --- |
|  | Your household population？（Household population ） |  |
|  | How many people of household population living at home in 6 months？ |  |
|  | How many people which are not household population living in your home for 6 months？（Include relatives, Nanny, et al） |  |
|  | （(Ask in rural areas) How many people of household population worked in other county in 6 months (Including accompanying persons, such as spouses, children and parents) |  |
|  | How many kilometers are there from the nearest medical institution to your home: ⑴<1km ⑵ 1- ⑶ 2- ⑷ 3- ⑸ 4- ⑹ 5km and above |  |
|  | How many minutes does it take from your home to your nearest medical institution? (The fastest way to get on foot or by car) |  |
|  | For general illnesses, what kind of medical institutions do your family usually choose for medical treatment:  ⑴ clinics / village health room ⑵ community health service stations ⑶ Township hospitals ⑷ community health service centers ⑸ general hospital ⑹ Chinese medicine hospital ⑺ others |  |
|  | Compared with 5 years ago, what the changes in your family’s convenience of seeing a doctor: ⑴ substantial improvement ⑵ slightly improved ⑶ no change ⑷ slightly worse ⑸ significantly worse |  |
|  | Compared to five years ago, what the changes in your family's cost of seeing a doctor : ⑴ great drop ⑵ Slight drop ⑶ No change ⑷ Slight increase ⑸ Increase significantly |  |
|  | What's the relationship do you think between the doctor and the patient?⑴ parents and children ⑵ teachers and students ⑶ friends ⑷ working partners ⑸ comrades ⑹ upper and lower ⑺ trading services ⑻ other |  |
|  | The most commonly used fuel for your home cooking is: ⑴ electricity ⑵ gas / natural gas / liquefied petroleum gas ⑶ biogas ⑷ kerosene ⑸ coal ⑹ firewood ⑺ other |  |
|  | Your drinking water type: ⑴ tap water ⑵ well water pumped by hand press ⑶ protected wells ⑷ rainwater collection ⑸ protected spring water ⑹ unprotected well water ⑺ unprotected spring water ⑻ truck or trolley water supply ⑼ surface water ⑽ other |  |
|  | Your home toilet type: ⑴ complete sewer water washed ⑵ dry toilet ⑶ triple biogas toilet ⑷ double urn funnel toilet ⑸ three grid septic tank ⑹ double pit alternating type ⑺ ventilation improved type ⑻ attic type ⑼ deep pond anti-freeze type ⑽ pit toilet with cover plate  ⑾ pit toilet without cover plate ⒀ no facilities or bushes or field ⒁ other |  |
|  | Your house type is: ⑴ a building of two or more storeys ⑵ brick cottage ⑶ adobe cottage ⑷ other |  |
|  | How many square meters of your home building area? |  |
|  | How much is your family's total income in last one year? (Urban households for the disposable income, rural households as a net income) |  |
|  | How much is your family’s total consumption expenditure in last one year? |  |
|  | How much is the expenditure on food？ |  |
|  | How much is the expenditure on clothing and daily necessities？ |  |
|  | How much is the expenditure on traffic and communication？ |  |
|  | How much is the expenditure on housing, utilities, and fuel？ |  |
|  | How much is the expenditure on education？ |  |
|  | How much is the expenditure on culture and entertainment？ |  |
|  | How much is the expenditure on drugs, medical services and supplies? |  |
|  | How much is the other expenditure? |  |
|  | Has your family been classified as a poor household? ⑴ yes ⑵ no |  |
|  | Is your home listed as a local minimum living security households ⑴ yes ⑵ no |  |
|  | If you are a poor or minimum security households, what do you think is the most important cause of economic hardship:  ⑴ less labor force ⑵ poor natural conditions or disasters ⑶ the ability to work was affected by disease damage ⑷ due to treatment of disease ⑸ unemployed ⑹ human factors (7) other |  |

Table 2. Family members' personal situation questionnaire

| A. Basic personal information | |  |  |  |  |  |  |
| --- | --- | --- | --- | --- | --- | --- | --- |
| The family member code (01 is the householder, the others self-coding in accordance with the order of investigation, the code can’t be changed once identified ) | | 01  户主 | 02 | 03 | 04 | 05 | 06 |
|  | Name： （01is the actual householder） |  |  |  |  |  |  |
|  | Relationship with householder：  ⑴householder ⑵spouse ⑶child ⑷son/daughter-in-law ⑸parents ⑹parent-in-law ⑺grandparents  ⑻grandchildren ⑼brother/sister  ⑽home service personnel ⑾other |  |  |  |  |  |  |
|  | Who answered the following question (judge by the investigator): (1) self ⑵ others |  |  |  |  |  |  |
|  | Household registration: ⑴ the local county / district ⑵ outside the local county / district ⑶ other provinces ⑷ undetermined |  |  |  |  |  |  |
|  | Nature of household registration: ⑴ agriculture ⑵ non-agricultural |  |  |  |  |  |  |
|  | Gender: ⑴ male ⑵ female |  |  |  |  |  |  |
|  | Nationalities: ⑴ Han ⑵ Zhuang ⑶ Hui ⑷ Uygur ⑸ Mongolian ⑹ Tibetan ⑺ Manchu ⑻ Miao ⑼ other |  |  |  |  |  |  |
|  | Date of birth: Year (fill in 4 digits, e.g.: 1998) |  |  |  |  |  |  |
|  | Month (fill in 2 digits, such as: 07) |  |  |  |  |  |  |
|  | Height (cm) |  |  |  |  |  |  |
|  | Weight (kg) |  |  |  |  |  |  |
| Family member code | | 01 | 02 | 03 | 04 | 05 | 06 |
|  | Have you participated in the Urban Employee Basic Medical Insurance? ⑴ yes ⑵ no |  |  |  |  |  |  |
|  | Have you participated in the Urban Resident Basic Medical Insurance? ⑴ yes ⑵ no |  |  |  |  |  |  |
|  | Have you participated in the New Rural Cooperative Medical Scheme? ⑴ yes ⑵ no |  |  |  |  |  |  |
|  | Have you participated in the Urban and Rural Resident Basic Medical Insurance? ⑴ yes ⑵ no |  |  |  |  |  |  |
|  | Have you purchased commercial health insurance? ⑴ yes ⑵ no |  |  |  |  |  |  |
|  | Have you taken other medical insurance? ⑴ yes ⑵ no |  |  |  |  |  |  |
|  | Are you a medical aid population for the government? ⑴ yes ⑵ no ⑶ unknown |  |  |  |  |  |  |
| Continue to ask the population aged 15 years and over (born before August 15, 1998), the population under the age of 15 transferred to Table 3 | | | | | | | |
|  | Marital status: ⑴ unmarried ⑵ married ⑶ widowed  ⑷ divorce ⑸ other |  |  |  |  |  |  |
|  | Educational status: ⑴ illiterate ⑵ primary school ⑶ junior high school ⑷senior high school ⑸ technical school ⑹ secondary school (in technology) ⑺ junior college ⑻ undergraduate and above |  |  |  |  |  |  |
|  | Employment status: (1) employed ⑵ retired ⑶ students ⑷ lost job ⑸ unemployed |  |  |  |  |  |  |
|  | Occupational type (answered by the employed and retired):  ⑴leader of organs, enterprises and institutions ⑵ professional and technical personnel ⑶ clerk and related personnel ⑷business/service industry personnel ⑸ productive personnel of agriculture, forestry, animal husbandry and fishery water production industry ⑹equipment operators of production and transport industry ⑺ military ⑻ other |  |  |  |  |  |  |
| B. Physical function | |  |  |  |  |  |  |
| Family member code | | 01 | 02 | 03 | 04 | 05 | 06 |
|  | Mobility：  ⑴ walk around, without any difficulties ⑵ action some inconvenience ⑶ can’t get out of bed activities |  |  |  |  |  |  |
|  | Self-care (toilet, dress up on the toilet, etc.):  ⑴ no problem ⑵ some problems  ⑶ can't go to toilet or wear clothes by oneself |  |  |  |  |  |  |
|  | Usual activities (e.g. work, read, housework)：  ⑴have no problems doing usual activities ⑵have some problem ⑶unable to do usual activities |  |  |  |  |  |  |
|  | Pain/discomfort：  ⑴have no pain or discomfort ⑵have moderate pain or discomfort ⑶have extreme pain or discomfort |  |  |  |  |  |  |
|  | Anxiety/depression：  ⑴consciously anxiety or depression ⑵ consciously moderate anxiety or depression ⑶ consciously extremely anxious or depressed |  |  |  |  |  |  |
|  | Please figure out the score that best describe your health today:  ├---┼—-┼—-┼—-┼—-┼—-┼—-┼—-┼—-┼—-┼—  0 10 20 　30 　40 　50 60 70 80 90 100  Worst health status best health status |  |  |  |  |  |  |
| C. Healthy behavior | |  |  |  |  |  |  |
| Family member code | | 01 | 02 | 03 | 04 | 05 | 06 |
|  | Your present smoking status:  ⑴ every day ⑵ not every day (3) non-smoker (skip to 60) |  |  |  |  |  |  |
|  | How old do you start smoking? (year) |  |  |  |  |  |  |
|  | How many cigarettes do you suck on average day in last one week? (Skip 61) |  |  |  |  |  |  |
|  | Your past smoking status:  ⑴ every day ⑵ not every day (3) non-smoker |  |  |  |  |  |  |
|  | Have you drank wine in last 12 months: ⑴ yes ⑵ no (skip to 64) |  |  |  |  |  |  |
|  | Your drinking frequency:  ⑴ at least 3 times a week ⑵ 1-2 times a week ⑶ less than once a week |  |  |  |  |  |  |
|  | c How many drinking unit do your every time average amount of alcohol drinking equal to (standard drinking unit)?  (Converted by investigator)  (50g white wine(40^o^ and above)= 2; 50g white wine (under 40^o^)= 1.5; 500g wine = 5; 1 bottle of beer = 2; 1 can of beer = 1; 500g rice wine = 6.5) |  |  |  |  |  |  |
|  | How many physical exercises do you have averaged per week in past six months :  ⑴ 6 times and above ⑵ 3-5 times ⑶ 1-2 times  ⑷ less than 1 ⑸ never exercise (skip to 67) |  |  |  |  |  |  |
|  | What's the average intensity of your per exercise (self-breathing, heartbeat to speed up the feeling):  ⑴ mild ⑵ moderate (3) severe |  |  |  |  |  |  |
|  | How long do you exercise per average time (minutes)? |  |  |  |  |  |  |
| Family member code | | 01 | 02 | 03 | 04 | 05 | 06 |
|  | Do you have a health record?  ⑴ yes ⑵ no ⑶ unknown |  |  |  |  |  |  |
|  | Do you have a healthy examination in the last 12 months? (Not including the etiological examination)  ⑴ yes ⑵ no |  |  |  |  |  |  |
|  | How many times do you brush your teeth per average day?:  ⑴ 2 and above ⑵ 1 ⑶ less than 1 ⑷ never |  |  |  |  |  |  |
| D. 慢性疾病 | |  |  |  |  |  |  |
|  | Have you been diagnosed with high blood pressure by a doctor?      ⑴ yes ⑵ no (skip to 75) |  |  |  |  |  |  |
|  | The frequency of your current use of antihypertensive drugs:  ⑴ by doctor's advice every day ⑵ occasionally or when necessary to take ⑶ never take |  |  |  |  |  |  |
|  | Your last time to measure blood pressure:  ⑴ within a week ⑵ within a month ⑶ within three months ⑷ within six months ⑸ six months ago |  |  |  |  |  |  |
|  | Are your current blood pressure normal? ⑴ yes ⑵ no ⑶ not clear |  |  |  |  |  |  |
|  | In the past three months, is there any medical staff to guide you on hypertension prevention and control?  ⑴ yes ⑵ no |  |  |  |  |  |  |
|  | Have you been diagnosed with diabetes by a doctor?    ⑴ yes ⑵ no (skip to 80) |  |  |  |  |  |  |
|  | The frequency of your current use of hypoglycemic drugs is:  ⑴ by doctor's advice every day ⑵ occasionally or when necessary to take ⑶ never take (skip to 78) |  |  |  |  |  |  |
| Family member code | | 01 | 02 | 03 | 04 | 05 | 06 |
|  | How do you use hypoglycemic drugs at present:  ⑴ oral ⑵ injection ⑶ both (1) and (2) |  |  |  |  |  |  |
|  | Your last time to measure blood sugar (including medical institution measurement and self-measurement):  ⑴ within a month (2) within three months (3) within six months (4) six months ago |  |  |  |  |  |  |
|  | Are your current blood glucose levels normal?    ⑴ yes ⑵ no ⑶ not clear |  |  |  |  |  |  |
|  | For the past six months, do you have other chronic diseases diagnosed by your doctor? *  ⑴ yes ⑵ no (skip to 84) |  |  |  |  |  |  |
|  | The first chronic disease (disease name)  (If there are many kinds of chronic diseases, fill in turns according to the severity of illness from high to low ) |  |  |  |  |  |  |
| 81A | Check and fill the first disease code |  |  |  |  |  |  |
|  | The second chronic disease (disease name) |  |  |  |  |  |  |
| 82A | Check and fill the second disease code |  |  |  |  |  |  |
|  | The third chronic disease (disease name) |  |  |  |  |  |  |
| 83A | Check and fill the third disease code |  |  |  |  |  |  |

* Note: Chronic disease is one of the following:

① The chronic disease was diagnosed clearly by doctor within 6 months before survey;② The chronic disease diagnosed by a doctor 6 months ago, recrudesced within 6 months and treated by medication, physical therapy and so on, or always receiving treatment to control the recurrence of the chronic disease.

| E. population health of 60 years and above (Answered by the population were born before August 15, 1953. Others transferred to Table 3) | | | | | | | |
| --- | --- | --- | --- | --- | --- | --- | --- |
| Family member code | | 01 | 02 | 03 | 04 | 05 | 06 |
|  | Your main economic source is: ⑴ own or spouse ⑵ children ⑶ grandchildren ⑷ relatives ⑸ friends ⑹ social relief ⑺ other |  |  |  |  |  |  |
|  | For the last six months, what is the situation of your walk:  ⑴ bed-ridden for long time, only can sit with other's help ⑵ can't walk without others' help ⑶ can't go out alone without others' help ⑷ walking freely |  |  |  |  |  |  |
|  | In the last 6 months, what is your hearing situation:  ⑴ difficult to hear clearly ⑵ need others to improve the sound ⑶ can hear clearly |  |  |  |  |  |  |
|  | Whether you have difficulty when you talking in the last 6 months? ⑴yes ⑵ no |  |  |  |  |  |  |
|  | Within six months, the degree of difficulty that you identify the acquaintance out of 20 meters away: (people with glasses answer the case of wearing glasses)  ⑴ no or mild difficulty ⑵ consciously moderate difficulties ⑶ consciously extremely difficult |  |  |  |  |  |  |
|  | Whether you need someone to take care of your daily life in the past 1 month? ⑴ yes ⑵ no |  |  |  |  |  |  |
|  | When you need to take care, who is the main person to help:  ⑴ spouse ⑵ children ⑶ grandchildren ⑷ brothers and sisters ⑸ relatives ⑹ neighbors ⑺ nanny ⑻ community ⑼ other ⑽ no one to help |  |  |  |  |  |  |

表3. A survey of the disease and injury within last two weeks

| Family member code | | 01 | 02 | 03 | 04 | 05 | 06 |
| --- | --- | --- | --- | --- | --- | --- | --- |
|  | Whether you have disease or injury within two weeks before the survey?  ⑴ yes (continue to ask the following questions) ⑵ no (transferred to Table 4) |  |  |  |  |  |  |

* Note: disease or injury within two weeks refers to one of the following condition:

within 14 days before the investigation: ① seeing a doctor, ② have medical treatment of the disease or injury(such as medication or massage, hot compress, and other auxiliary therapy), ③ due to illness , suspend one's working, schooling or bed-ridden one day and above (the elderly with obvious spirit sluggish, loss of appetite or infants and young children with abnormal crying, loss of appetite, etc.)

The following question answered by members with illness or injury within the two weeks before the investigation, and the investigators fill in order from the first column. Such as suffering from two or more disease, each disease need to ask, and each disease fill in a column, the member code unchanged.

| Family member code | |  |  |  |  |  |  |  |  |
| --- | --- | --- | --- | --- | --- | --- | --- | --- | --- |
|  | What illness or injury do you have? (Fill in the name) |  |  |  |  |  |  |  |  |
| 92A | （check and fill the disease code) |  |  |  |  |  |  |  |  |
|  | When did you get sick or injured?  ⑴ first broke out within two weeks ⑵ acute disease broke out two weeks ago (3) chronic disease continued within two weeks |  |  |  |  |  |  |  |  |
|  | How many days the illness lasted within two weeks (up to 14 days)? |  |  |  |  |  |  |  |  |
|  | How many days bed-ridden due to the illness within two weeks (up to 14 days)? (fill in 0 if none) |  |  |  |  |  |  |  |  |
|  | How many days do you suspend of working due to the illness (up to 14 days)? (fill in 0 if none) |  |  |  |  |  |  |  |  |
|  | How many days do you suspend of schooling due to the illness (up to 14 days)? (fill in 0 if none) |  |  |  |  |  |  |  |  |
|  | Whether you seen a doctor due to the illness within two weeks? ⑴ yes (skip to 100) ⑵ no |  |  |  |  |  |  |  |  |
| Family member code | |  |  |  |  |  |  |  |  |
|  | The reason for not to see a doctor within two weeks (single selection): (after asked the question, skip to 116)  ⑴ continue treating according to the doctor's advice after seeing doctor two weeks ago  ⑵ self-feel of disease is light ⑶ economic difficulties ⑷ inconvenient to see a doctor ⑸ no time  ⑹ traffic inconvenience ⑺ no effective measures ⑻ other reasons |  |  |  |  |  |  |  |  |
|  | How many times have you seen a doctor due to this illness within two weeks(times)？ |  |  |  |  |  |  |  |  |
|  | What kind of medical institutions were you first visited for this disease? :  ⑴clinic/village health room (2)community health service station (3) township hospital (4) community health service center (5) county-level health institutions (6)city-level health institutions (7)province-level, and above health institutions (8) other |  |  |  |  |  |  |  |  |
|  | How about the length of time spent on the waiting for the visiting:  ⑴ very short ⑵ short ⑶ general ⑷ long ⑸ very long |  |  |  |  |  |  |  |  |
|  | How about the environment for this visit:  ⑴ excellent ⑵ good ⑶ general ⑷ poor ⑸ bad |  |  |  |  |  |  |  |  |
|  | What do you think of the attitude of the health care provider to explain the problem to you :  ⑴ excellent ⑵ good ⑶ general ⑷ poor ⑸ bad |  |  |  |  |  |  |  |  |
|  | What do you think of the clarity of the treatment program that medical staff explained to you :  ⑴ excellent ⑵ good ⑶ general ⑷ poor ⑸ bad |  |  |  |  |  |  |  |  |
|  | How about the concern from medical staff when you state your conditions?  ⑴ excellent ⑵ good ⑶ general ⑷ poor ⑸ bad |  |  |  |  |  |  |  |  |
|  | How about your trust to the medical staff who were diagnosing and treating your disease:  ⑴ trust very much ⑵ trust ⑶ general ⑷ distrust ⑸ distrust very much |  |  |  |  |  |  |  |  |
|  | What do you think of the cost of this visit:  ⑴ not expensive ⑵ general ⑶ expensive |  |  |  |  |  |  |  |  |
|  | What is your overall satisfaction with this visit:  ⑴ satisfied (skip to 111) ⑵ general (skip to 111) ⑶ not satisfied |  |  |  |  |  |  |  |  |
|  | If you were not satisfied, what is your most dissatisfied: (single selection)  ⑴low level of technology(2)poor equipment conditions⑶limited variety of drugs⑷poor service attitude⑸charges unreasonable ⑹high medical costs ⑺fussy procedure⑻wait time too long⑼poor environmental conditions⑽provide unnecessary services (including drugs and examination) ⑾other |  |  |  |  |  |  |  |  |
|  | Whether you received an infusion treatment for the disease within two weeks? ⑴ yes ⑵ no |  |  |  |  |  |  |  |  |
|  | How much did you pay by yourself for the disease within two weeks? (Excluding reimbursement, and expenditure in personal medical accounts) |  |  |  |  |  |  |  |  |
|  | How many transportation costs and other related expenses (yuan) have been spent in the two weeks for the disease? |  |  |  |  |  |  |  |  |
|  | Within two weeks, did you visit a doctor of Chinese medicine hospital due to this disease? ⑴ yes ⑵ no |  |  |  |  |  |  |  |  |
|  | Within two weeks, whether you have been to the department of traditional Chinese medicine of the general Hospital due to this disease? ⑴ yes ⑵ no |  |  |  |  |  |  |  |  |
|  | Did you have self-treatment within two weeks?  ⑴ yes ⑵ no (skip to 120) |  |  |  |  |  |  |  |  |
|  | Did you use drugs in your self-treatment? ⑴ yes ⑵ no (transferred to Table 4) |  |  |  |  |  |  |  |  |
|  | Where did you get your self-treat medicine: (up to two)  ⑴ bought within two weeks (2) had in home (3) other |  |  |  |  |  |  |  |  |
|  |  |  |  |  |  |  |  |  |  |
|  | If the drugs was bought within two weeks, how much did you pay (yuan)?(Excluding reimbursement, and expenditure in personal medical accounts) |  |  |  |  |  |  |  |  |
|  | Whether you have taken the Chinese medicine for the disease within two weeks? ⑴yes ⑵ no |  |  |  |  |  |  |  |  |

Table 4. Survey of hospital status in the previous year

| Family member code | | 01 | 02 | 03 | 04 | 05 | 06 |
| --- | --- | --- | --- | --- | --- | --- | --- |
|  | For the past 12 months, did you have the situation that you have been diagnosed with hospitalization but you were not hospitalized?  ⑴ yes ⑵ no (skip to 124) |  |  |  |  |  |  |
|  | How many times? (With a disease doctor repeatedly diagnosed, counted as 1) |  |  |  |  |  |  |
|  | The reason why you should hospitalized but not hospitalized at the last time:  ⑴ no need ⑵ no effective treatment ⑶ economic difficulties ⑷ poor hospital service ⑸ no time ⑹ no bed ⑺ other |  |  |  |  |  |  |
|  | Nearly 12 months, whether you have hospitalized due to illness, physical examination, childbirth and other reasons?  ⑴yes ⑵ no (transferred to Table 5) |  |  |  |  |  |  |
|  | If hospitalized, how many times did you have (times)? |  |  |  |  |  |  |

The following information is answered by the members hospitalized within one year before the survey, fill in order by the investigators from the first column. If the number of hospitalizations for 2 times and above, the information of every time hospitalization should be ask. The information of every time hospitalization fill a column. The member code unchanged.

| Family member code | |  |  |  |  |  |  |
| --- | --- | --- | --- | --- | --- | --- | --- |
|  | Your reasons for this hospitalization: ⑴disease ⑵injury poisoning ⑶rehabilitation ⑷family planning ⑸childbirth ⑹health examination ⑺other |  |  |  |  |  |  |
|  | What was your illness or injury? (fill in the name of disease) |  |  |  |  |  |  |
| 127A | (Check and fill the first disease code) |  |  |  |  |  |  |
|  | The hospital admission time: (years) (fill in 4 digits, such as: 1998) |  |  |  |  |  |  |
|  | (Month) (fill in 2 digits, such as: 07) |  |  |  |  |  |  |
|  | What kind of medical institutions were you hospitalized:  ⑴ township hospital ⑵ community health service center  ⑶ county-level health institutions  ⑷ city-level health institutions  ⑸ province-level, and above health institutions (6) other |  |  |  |  |  |  |
|  | How about the utilization of Chinese medicine services for this hospitalization:  ⑴ inpatient institutions are Chinese medicine hospital  ⑵ inpatient institutions are department of Chinese medicine of general hospital ⑶ non-Chinese medicine |  |  |  |  |  |  |
|  | The time of waiting for hospitalization (day of admission) (days). |  |  |  |  |  |  |
|  | Have you done surgery during this hospitalization? ⑴yes ⑵ no |  |  |  |  |  |  |
|  | The number of days of this hospitalization (days). |  |  |  |  |  |  |
|  | Were there anyone nursing you during hospitalization?  ⑴ yes ⑵ no |  |  |  |  |  |  |
|  | If you have been nursed, the main caregiver is:  ⑴ spouse ⑵ children ⑶ parents ⑷ grandchildren ⑸ brothers and sisters ⑹ relatives ⑺ friends ⑻ nanny ⑼ nursing workers ⑽ other |  |  |  |  |  |  |
|  | This hospital discharge was due to: ⑴ healed up and doctor required ⑵ disease wasn't cured but doctor required ⑶ own requirements ⑷ other reasons |  |  |  |  |  |  |
|  | If you ask for hospital discharge, the reason is:  (1) obstinate illness ⑵ think oneself healed up⑶ economic difficulties ⑷ spent too much ⑸ poor hospital facilities ⑹ poor service attitude ⑺ poor doctor technology ⑻ other |  |  |  |  |  |  |
|  | What is the total cost of this hospitalization? |  |  |  |  |  |  |
|  | How much have you paid by yourself?  (Excluding reimbursement, and expenditure in personal medical accounts) |  |  |  |  |  |  |
| Family member code | |  |  |  |  |  |  |
|  | What is the total cost of this hospitalization, including traffic, accommodation, food, attendant and other expenses? (fill in 0 if none) |  |  |  |  |  |  |
|  | What do you think of the hospital ward environment?  ⑴excellent ⑵ good ⑶ general ⑷ poor ⑸ bad |  |  |  |  |  |  |
|  | What do you think of the attitude of the health care provider to explain the problem to you :  ⑴ excellent ⑵ good ⑶ general ⑷ poor ⑸ bad |  |  |  |  |  |  |
|  | What do you think of the clarity of the treatment program that medical staff explained to you :  ⑴ excellent ⑵ good ⑶ general ⑷ poor ⑸ bad |  |  |  |  |  |  |
|  | How about the concern from medical staff when you state your conditions?  ⑴ excellent ⑵ good ⑶ general ⑷ poor ⑸ bad |  |  |  |  |  |  |
|  | How about your trust to the medical staff who were treating your disease:  ⑴ trust very much ⑵ trust ⑶ general ⑷ distrust ⑸ distrust very much |  |  |  |  |  |  |
|  | What did you think of the hospital's medical expenses:  ⑴ not expensive ⑵ general ⑶ expensive |  |  |  |  |  |  |
|  | What was your overall satisfaction with this hospital:  ⑴ satisfied (transfer to Table 5) ⑵ general (transferred to Table 5) ⑶ dissatisfied |  |  |  |  |  |  |
|  | If you were not satisfied, what is your most dissatisfied: (single selection)  ⑴ low level of technology (2) poor equipment conditions ⑶ limited variety of drugs ⑷ poor service attitude ⑸ charges unreasonable ⑹ high medical costs ⑺ fussy procedures ⑻ wait time too long ⑼ poor environmental conditions ⑽ provide unnecessary services (including drugs and examination) ⑾ other |  |  |  |  |  |  |

Table 5. Children under 5 years of age questionnaire

This table is answered by the child's mother or insider (investigating children were born after August 15, 2008

| Family member code | | 01 | 02 | 03 | 04 | 05 | 06 |
| --- | --- | --- | --- | --- | --- | --- | --- |
|  | The following questions are answered by: (Investigators choose) ⑴ mother ⑵ father ⑶ other members of the family |  |  |  |  |  |  |
|  | Who usually live with children:  ⑴ parents ⑵ grandparents ⑶ brother / sister ⑷ relatives ⑸ other |  |  |  |  |  |  |
|  | (Ask in rural areas) Does the child's father have long been working in the field? ⑴ yes ⑵ no |  |  |  |  |  |  |
|  | (Ask in rural areas) Does the child's mother have long been working in the field? ⑴ yes ⑵ no |  |  |  |  |  |  |
|  | Has the child ever eaten breast milk? ⑴ yes ⑵ no (skip to 157) |  |  |  |  |  |  |
|  | How long did the children rely on breastfeeding (months)? (fill in 0 if none) |  |  |  |  |  |  |
|  | Which month the children began add supplementary food regularly (month)? |  |  |  |  |  |  |
|  | Nearly within 12 months, how many times the child received health examination (times)? (Not including examinations for the treatment of the disease)  (fill in 0 if none and skip to 160) |  |  |  |  |  |  |
|  | Whether measured weight in health examination? ⑴ yes ⑵ no |  |  |  |  |  |  |
|  | Whether measured height in health examination? ⑴ yes ⑵ no |  |  |  |  |  |  |
|  | Do children have the inoculate card? ⑴ yes⑵ no ⑶ unknown |  |  |  |  |  |  |
| Family member code | | 01 | 02 | 03 | 04 | 05 | 06 |
| Check the vaccination card and fill in the table according to the record, ask the inoculate situation when there is no card | |  |  |  |  |  |  |
|  | Whether your child inoculated the BCG vaccine？ ⑴yes ⑵no |  |  |  |  |  |  |
|  | How many times did your child inoculate the DTP vaccine (times)? |  |  |  |  |  |  |
|  | How many times did your child inoculate the polio vaccines (sugar pills) (times)? |  |  |  |  |  |  |
|  | How many times did your child inoculate the measles-containing vaccines (including MR, MMR, MM, and Measles)? (Times) |  |  |  |  |  |  |
|  | How many times did your child inoculate the hepatitis B vaccine (times)? |  |  |  |  |  |  |
|  | How satisfied were you with the provision of vaccination services for your child by the healthcare institution? :  ⑴ satisfied ⑵ general ⑶ not satisfied |  |  |  |  |  |  |

Table 6. 15-64-year-old women's questionnaire

Answered by the women were born on August 16, 1949 to August 15, 1998

| 被调查成员代码 | | 01 | 02 | 03 | 04 | 05 | 06 |
| --- | --- | --- | --- | --- | --- | --- | --- |
|  | For the past 12 months, have you ever done a gynecological examination? ⑴ yes ⑵ no |  |  |  |  |  |  |
|  | For the past 12 months, have you done a cervical smear examination? ⑴ yes ⑵ no |  |  |  |  |  |  |
|  | For the past 12 months, have you done a breast examination? ⑴ yes ⑵ no |  |  |  |  |  |  |
|  | (Ask in rural areas) Whether you can get if you want to be serviced by a female doctor in a township health center?  ⑴ yes ⑵ no ⑶ never thought ⑷ unknown |  |  |  |  |  |  |
|  | (Ask in rural areas) Does your husband working long-term in the field? ⑴ yes ⑵ no |  |  |  |  |  |  |
|  | How many times have you been pregnant? (fill in 0 if none and end the survey) |  |  |  |  |  |  |
|  | How many children did you give birth (people)? (fill in 0 if none and end the survey) |  |  |  |  |  |  |
|  | The time of your last time of childbirth: (years) (fill in 4 digits, such as: 1998) |  |  |  |  |  |  |
|  | (Month) (fill in 2 digits, such as: 07) |  |  |  |  |  |  |
| The following questions answered by the women who have childbirth after August 15, 2008 | | | | | | | |
|  | Your last child's gender: ⑴ male ⑵ female |  |  |  |  |  |  |
|  | How many prenatal checks (times) have you made during the period your last pregnant? (fill in 0 if none, skip to 182 questions) |  |  |  |  |  |  |
| Family member code | | 01 | 02 | 03 | 04 | 05 | 06 |
|  | Whether you done blood tests in the prenatal examination after pregnant? ⑴ yes ⑵ no |  |  |  |  |  |  |
|  | Did you measure blood pressure? ⑴yes ⑵ no |  |  |  |  |  |  |
|  | Have you checked urine? ⑴ yes ⑵ no |  |  |  |  |  |  |
|  | Have you done a type-B ultrasonic check? ⑴ yes ⑵ no |  |  |  |  |  |  |
|  | Type of childbirth: ⑴ natural childbirth (skip to 184 questions) ⑵ cesarean section |  |  |  |  |  |  |
|  | If you giving birth by cesarean section, who is mainly proposed: ⑴ own requirements ⑵ doctor recommendations ⑶ other people suggested |  |  |  |  |  |  |
|  | Where are you giving birth:  ⑴county and above hospitals ⑵ maternal and child health care institutions ⑶ township street hospitals ⑷ community health service center ⑸ clinic/ health center / health station ⑹ home ⑺ other |  |  |  |  |  |  |
|  | If you giving birth at home, who is the midwife: ⑴ the doctor at the township or above ⑵ village doctor ⑶ professional midwife ⑷ non-professional midwife ⑸ family member ⑹ other |  |  |  |  |  |  |
|  | What is the weight of a child at birth? |  |  |  |  |  |  |
|  | What is the total cost of childbirth? |  |  |  |  |  |  |
|  | How much do you pay by yourself? (fill in 0 if none)  (Excluding reimbursement, and expenditure in personal medical accounts) |  |  |  |  |  |  |
|  | How many times of postpartum visit do you receive within 42 days postpartum? (None filled with 0) |  |  |  |  |  |  |

After all the household members have completed the investigation, fill in the following table (the result is filled in the right column)

| 1 | Number of residential population of this household in the last 6 months(person) |  |
| --- | --- | --- |
| 2 | 15-64 years old number of women (person) |  |
| 3 | Number of children under 5 years old (person) |  |
| 4 | Number of people suffering disease or injury in last two weeks(person) |  |
| 5 | Number of people hospitalized in the previous year (person) |  |
| 6 | The household is: ⑴ first-time investigation ⑵ re-investigation |  |
| 7 | The household is: ⑴ household which is first sampled ⑵ substitute investigation households |  |
| 8 | Survey duration (minutes) |  |
